# Targeting key pathways in tumour growth and development

**DOI:** 10.1038/sj.bjc.6602601

**Published:** 2005-05-31

**Authors:** R S Herbst

**Affiliations:** 1 Department of Thoracic/Head and Neck Medical Oncology, The University of Texas MD Anderson Cancer Center, 1515 Holcombe Boulevard, Unit 432, Houston, TX 77030-4009, USA

Identification of a range of targets essential and specific for tumour progression has resulted in a new generation of anticancer agents, the molecular-targeted therapies. Of these, agents that inhibit specific growth factors, such as epidermal growth factor (EGF) and vascular endothelial growth factor (VEGF), have shown particular promise and are now available for use in patients. In this supplement we explore the potential of molecular-targeted agents and highlight specific considerations as an increasing number are clinically approved.

In the first of these articles, *Roy Bicknell* reviews the key molecular targets for novel antitumour agents and the challenge of translating the early promise of this new class of drugs into clinical practice. The wealth of *in vitro* and *in vivo* data obtained with ZD6474, a selective inhibitor of VEGF and EGF receptor tyrosine kinase activity, is reviewed by *Anderson Ryan* and *Stephen Wedge*. The clinical evaluation of ZD6474 to date is summarised by *John V Heymach*, who highlights the successes of the Phase I studies and discusses the ongoing Phase II programme, which aims to evaluate the clinical value of this agent alone and in combination with certain chemotherapy regimens.

I have elected to review the role of novel targeted therapies in the clinic, their current use, potential toxicity issues and finally what I have called the *What*?, *When? How? and Who*? of targeted therapy. As such, I attempt to distil our current knowledge and discuss how we might use these agents to best effect. Finally, *Malcolm Ranson* and *Gordon Jayson* consider the future of targeted therapy, highlighting the need for closer integration of preclinical and clinical data, a greater use of genomic and proteomic techniques, and further refinement of clinical trial design.

